# C24:0 and C24:1 sphingolipids in cholesterol-containing, five- and six-component lipid membranes

**DOI:** 10.1038/s41598-020-71008-8

**Published:** 2020-08-24

**Authors:** Emilio J. González-Ramírez, Aritz B. García-Arribas, Jesús Sot, Félix M. Goñi, Alicia Alonso

**Affiliations:** 1 Instituto Biofisika (CSIC, UPV/EHU), 48940 Leioa, Bilbao, Basque Country Spain; 2 Departamento de Bioquímica, University of the Basque Country (UPV/EHU), 48940 Bilbao, Spain

**Keywords:** Biophysics, Biological fluorescence, Membrane biophysics, Membrane structure and assembly, Atomic force microscopy

## Abstract

The biophysical properties of sphingolipids containing lignoceric (C24:0) or nervonic (C24:1) fatty acyl residues have been studied in multicomponent lipid bilayers containing cholesterol (Chol), by means of confocal microscopy, differential scanning calorimetry and atomic force microscopy. Lipid membranes composed of dioleoyl phosphatidylcholine and cholesterol were prepared, with the addition of different combinations of ceramides (C24:0 and/or C24:1) and sphingomyelins (C24:0 and/or C24:1). Results point to C24:0 sphingolipids, namely lignoceroyl sphingomyelin (lSM) and lignoceroyl ceramide (lCer), having higher membrane rigidifying properties than their C24:1 homologues (nervonoyl SM, nSM, or nervonoyl Cer, nCer), although with a similar strong capacity to induce segregated gel phases. In the case of the lSM-lCer multicomponent system, the segregated phases have a peculiar fibrillar or fern-like morphology. Moreover, the combination of C24:0 and C24:1 sphingolipids generates interesting events, such as a generalized bilayer dynamism/instability of supported planar bilayers. In some cases, these sphingolipids give rise to exothermic curves in thermograms. These peculiar features were not present in previous studies of C24:1 combined with C16:0 sphingolipids. Conclusions of our study point to nSM as a key factor governing the relative distribution of ceramides when both lCer and nCer are present. The data indicate that lCer could be easier to accommodate in multicomponent bilayers than its C16:0 counterpart. These results are relevant for events of membrane platform formation, in the context of sphingolipid-based signaling cascades.

## Introduction

Sphingolipids are important structural membrane lipids, as well as second messengers in diverse cellular signaling cascades^1–4^. Among the sphingolipid subfamilies, ceramides (Cer) have received the attention of investigators due to their particular biophysical properties and their role in apoptotic processes^5–8^. Cer are also important in the *stratum corneum* of the skin^9^. The biophysical properties of Cer cause dramatic changes in membranes, as this sphingolipid tends to induce the formation of segregated areas (‘domains’), giving rise to bilayer lateral heterogeneity^10–13^. In turn the presence of domains has an impact on the diffusivity of proteins and their capacity to cluster and exert their physiological effects^14–16^. Mammals have 6 ceramide synthases, of which CerS5 predominantly produces C16:0 Cer, while CerS2 yields ceramides incorporating C22-C24 fatty acids^17^. For long and very-long chain Cer (longer than 14 carbons in their N-acyl group), the effects on membranes may also include lipid trans-bilayer motion, or flip-flop^18^ (therefore affecting bilayer asymmetry) and a notable increase in solute permeability across the membrane^19,20^, which may decisively distort cell homeostasis. Most of the experiments classically performed with model membranes have been focused on C16:0 ceramide (pCer), as it is a simple, saturated long-chain ceramide and, more importantly, C16:0 sphingolipids (and particularly C16:0 sphingomyelin, pSM) are among the most abundant sphingolipid species in cells^21,22^. Cer can be synthesized through a number of metabolic routes^23^, but the more prevalent pathway in signaling cascades is the action of sphingomyelinases that cleave the headgroup of sphingomyelin and convert it directly to Cer^24^. Thus, there is a plethora of sphingolipid species, but most Cer studies have been centered on pCer, perhaps due to the abundance of the respective pSM.

However, more recent studies in the field of lipidomics have pointed out the importance of sphingolipids containing N-acyl chains such as C24:1 and C24:0 in many cell lines^22,25,26^. A recent report from our laboratory^22^, included a rather extensive collection of lipidomics data, original or previously published, showing that in the vast majority of mammalian cells (with the exception of brain tissue) the most abundant Cer species were those containing C24:0 and C24:1 in their N-acyl chains, while the main species in SM was C16:0. The very-long chain (VLC) Cer (20–24 carbon atoms in their N-acyl chain) appear to have specific properties at the cell level. Law et al.^27^ found that VLC Cer were toxic and induced mithophagy in cardiomyocytes. Studies on phagocytes^28^ revealed that VLC Cer could be essential for phagosome maturation in immune cells. VLC sphingolipids were found to be crucial in the survival of invariant natural killer T cells in thymus and liver^29^. At the biophysical level there are interesting studies on the properties of VLC sphingolipids dispersed in pure form in aqueous media^30–35^, but not many reports on their biophysical effects in multicomponent membranes^30,36,37^. The importance of multicomponent membrane studies is increased when one of the lipids is cholesterol (Chol), not only because of the high prevalence of Chol in most animal membranes (25–50 mol% of total lipids depending on the cell line and subcellular organelle^26,38,39^) but also due to the particular properties that Chol confers to the membrane. Both Chol and Cer are highly hydrophobic molecules that tend to occupy the spaces between the lipid acyl chains, however Cer give rise to highly packed and stoichiometrically constant gel phases in the Cer-enriched domains^40^, while Chol has a tendency to mix with phospholipids and induce ‘liquid-ordered’ (L_o_) phases, sometimes at the nano scale^41,42^. L_o_ phases are often considered as a middle ground between a fluid phase and a gel phase in terms of molecular packing and nanomechanical resistance^42–45^. Both Cer and Chol tend to compete for the same interaction sites, although at high concentrations of both Chol and Cer a different kind of stable gel phase can be observed, stabilized by 3-way interactions between Chol, Cer and a high-melting bilayer-forming lipid (such as DPPC or SM)^40,46^. This kind of phases has also been observed by adding ceramide to cell lipid extracts, thus giving rise to non-stoichiometrically constant gel phases^47^. These reports point to Cer-Chol interactions as potential regulators for Cer-related signaling cascades^15,17,19,48,49^ but, while there are some in vivo experiments indicating the importance of Cer-rich domains in cell death^50,51^, confirmation of the Cer-Chol hypothesis is severely hampered by the large amount of components present in those cell signaling cascades and, more specifically, in cell-death processes.

In a previous report^37^, we explored the effects of C24:1 sphingolipids in multicomponent bilayers containing Chol and C16:0 sphingolipids. Our findings revealed an interesting impact of the presence of C24:1 sphingolipids as their stiffening effect on lipid membranes was much lower than their C16:0 counterparts and, more importantly, in some cases they were capable of coexisting in a single gel phase while, in other mixtures, they showed a tendency to displace each other and form separate gel phases. This complex behavior of the C24:1sphingolipids could be due to either the longer N-acyl chain or the presence of a C=C bond. In the present study, we have studied the effect of the C=C bond in the N-acyl chain by comparing sphingolipids of the same chain length, C24:0 and C24:1. We have employed the same techniques and experimental approach as in our previous report, differential scanning calorimetry (DSC), confocal fluorescence microscopy and atomic force microscopy (AFM), well established in the field of membrane biophysics. The mixtures under study were model membranes composed of DOPC:SM:Chol (2:1:1) + 30 mol% Cer, in which SM and Cer were either C24:0, or lignoceroyl (respectively lSM and lCer), C24:1, or nervonoyl (respectively nSM and nCer), or an equimolecular mixture of the saturated and unsaturated forms. The complexity of 5- and 6-component mixtures makes impractical their description in terms of phase diagrams. This does not impede attaining reliable conclusions concerning the C24 sphingolipids. Some of the systems under study reached easily their equilibrium conditions. However, in planar bilayers containing both C24:0 and C24:1, dynamic (unstable) mixtures were observed where gel phases (domains) tended to disappear over time, at variance with our previous results obtained with C16:0 and C24:1 sphingolipids.

## Results

The results in this study reflected the capacity of our biophysical techniques to characterize multicomponent model lipid bilayers in the presence of lateral heterogeneity, e.g. rigid (less fluid) domains in a more fluid continuous phase. The use of a lipid fluorophore (RhoPE) was required for confocal fluorescence microscopy; this particular probe tended to partition away from the domains, into the continuous phase. Thus, domains appeared in fluorescence images as dye-depleted, non-stained (or poorly stained) areas. Note, however, that the partition properties of the fluorescent reporters used in this work may not be uniquely related to specific thermodynamic phases, particularly when complex mixtures are used, thus the fluorescent probe information should be interpreted in combination with the other techniques. Particularly rigid gel phases could also be identified by DSC as they have a melting temperature (T_m_) in the 20–100 °C range, giving clearly distinguishable endothermic signals in the calorimetric experiment. Besides, AFM experiments provided information about the topography and shape of the domains (imaging mode) and the nanomechanical resistance of each phase present (force spectroscopy mode). Finally, a comparative assessment of the different results for each sample provided an idea on how each lipid affected the multicomponent system.

### Preliminary experiments: samples without ceramide

**Figure 1:**
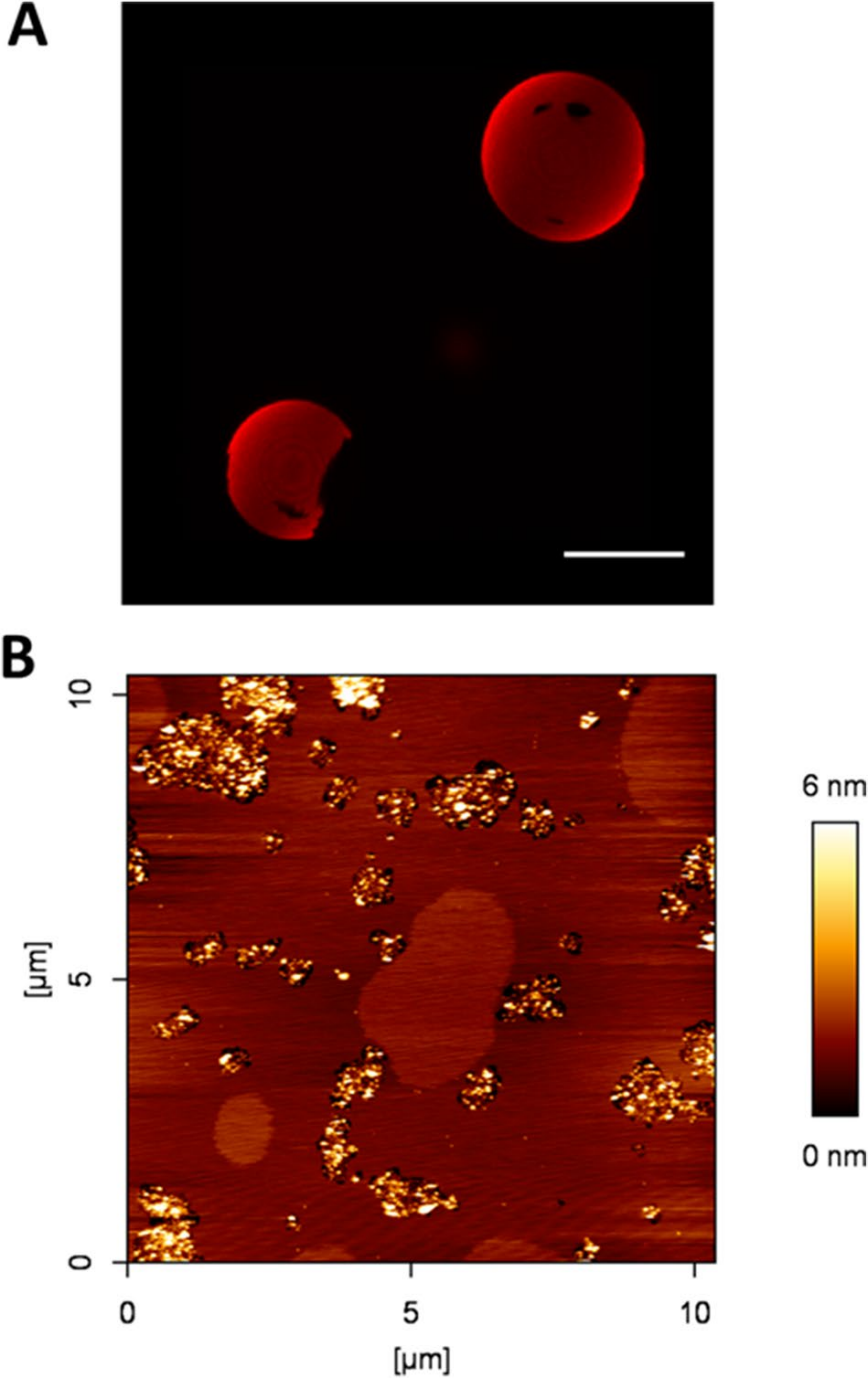
Topographical characterization of DOPC:lSM:nSM:Chol (2:0.5:0.5:1). Confocal microscopy 3D (z-stack) image of RhoPE-stained GUVs (**A**) and AFM imaging of SPBs (**B**). Scale bar in A: 10 μm. Temperature was 23 ± 1 °C.

In order to properly understand the effects of C24:0 and C24:1 ceramides in the lipid bilayers, it was first needed to characterize the system in the absence of any Cer. The presence of liquid-ordered domains had been checked for DOPC:lSM:Chol (2:1:1) and DOPC:nSM:Chol (2:1:1) samples in a previous study^24^, with the former, but not the latter, exhibiting domains. (Note that lipid compositions are given as mole ratios throughout this paper). However, the DOPC:lSM:nSM:Chol (2:0.5:0.5:1) sample had not been studied previously. The importance of this preliminary test is based on the fact (reported as well in Maté et al.^25^) that nSM is able to override the presence of domains in DOPC:pSM:nSM:Chol (2:0.5:0.5:1), which could resemble our test since both pSM and lSM are saturated sphingomyelins. However, our results indicate that, contrary to our predictions, DOPC:lSM:nSM:Chol showed clear segregated domains in both AFM and confocal fluorescence experiments (Fig. 1). These domains looked rather unstable in the SPBs scanned by AFM, sometimes appearing or disappearing over time or as a function of temperature in the form of slightly more clear areas over a brown background (Supp. Fig. S1), while in GUVs the domains appeared to be in equilibrium. This will be further commented on in the “Discussion” section. The nanomechanical resistance (i.e. breakthrough force) detected for the Cer-free sample was the same for both phases present (9.1 ± 1.4 nN). The similar properties of these phases and their time-dependent evolution might point to a cluster-like behavior (one-phase non-random mixing) rather than proper domain separation^52,53^, although this hypothesis would require appropriate experimental testing.

### lSM-based samples

**Figure 2:**
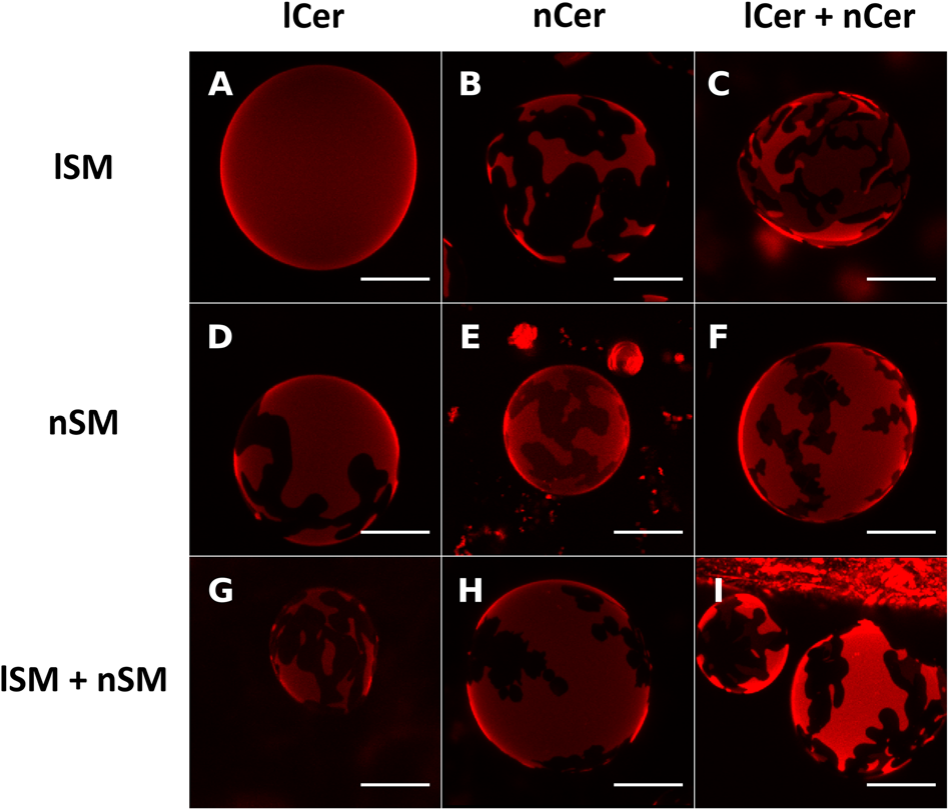
Confocal microscopy 3D (z-stack) images of fluorescent giant unilamellar vesicles containing 0.4% RhoPE. DOPC:lSM:Chol (2:1:1) + 30% lCer (**A**), + 30% nCer (**B**), + 15% lCer & 15% nCer (**C**); DOPC:nSM:Chol (2:1:1) + 30% lCer (**D**), + 30% nCer (**E**), + 15% lCer & 15% nCer (**F**); DOPC:lSM:nSM:Chol (2:0.5:0.5:1) + 30% lCer (**G**), + 30% nCer (**H**), + 15% lCer and 15% nCer (**I**). Scale bars: 10 μm. Temperature was 23 ± 1 °C.

**Figure 3:**
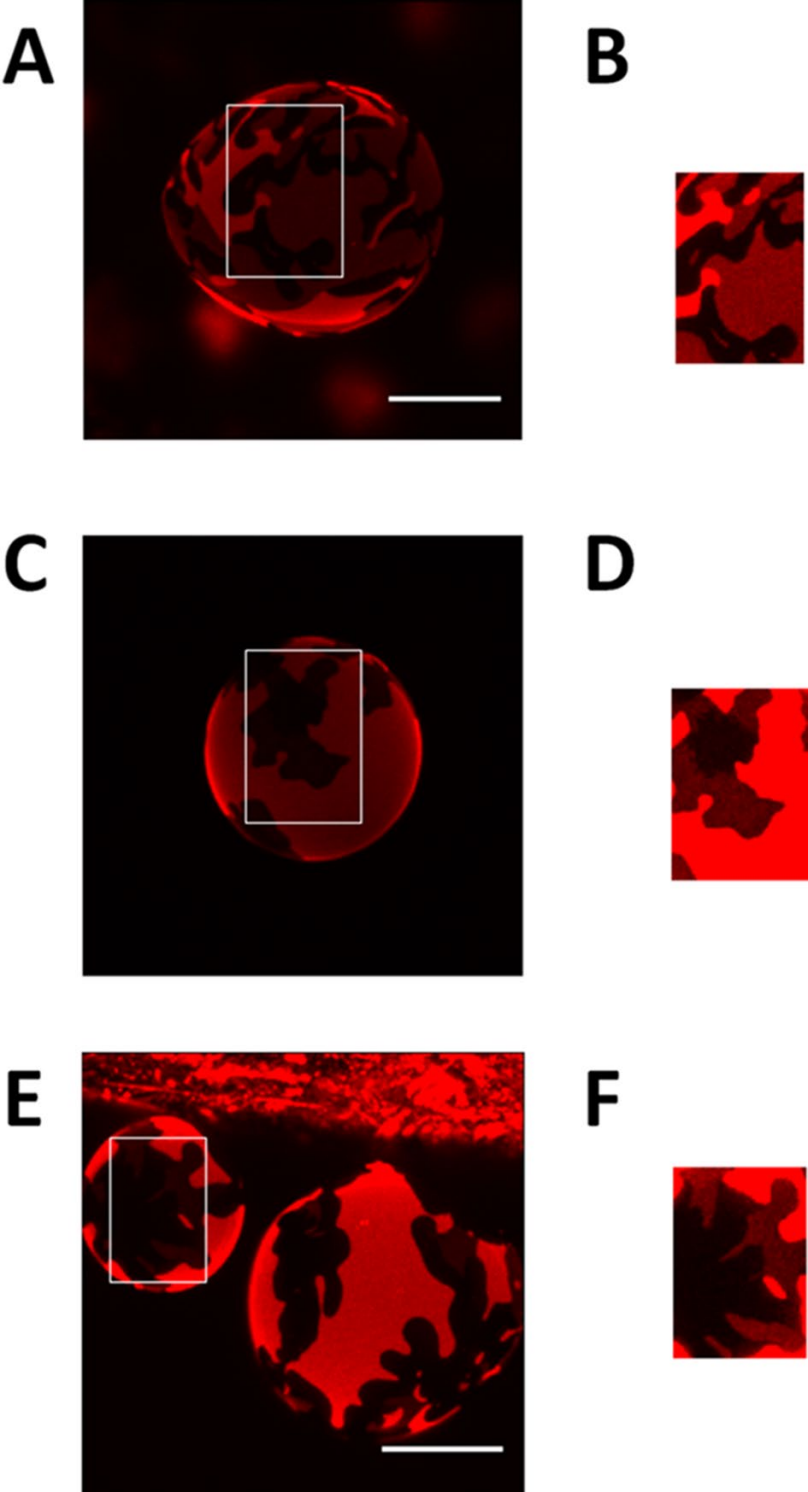
Confocal microscopy 3D (z-stack) zoom-in images of fluorescent giant unilamellar vesicles containing 0.4% RhoPE. DOPC:lSM:Chol (2:1:1) + 15% lCer & 15% nCer (**A**, as depicted in Fig. 2c) and zoom-in of the fluorescence-depleted area (**B**), DOPC:nSM:Chol (2:1:1) + 15% lCer and 15% nCer (**C**, as depicted in Fig. 2f) and zoom-in of the fluorescence-depleted area (**D**); DOPC:lSM:nSM:Chol (2:0.5:0.5:1) + 15% lCer and 15% nCer (E, as depicted in Fig. 2i) and zoom-in of the fluorescence-depleted area (**F**). Scale bars: 10 μm. Temperature was 23 ± 1 °C.

**Figure 4:**
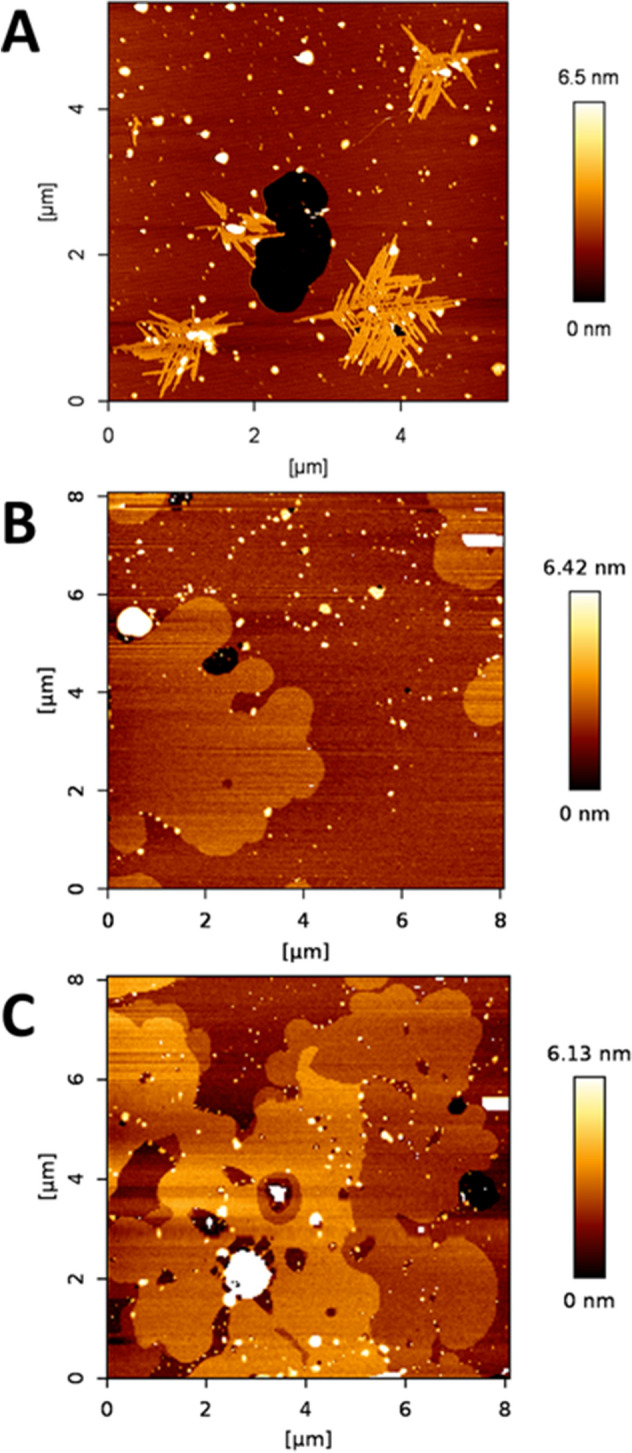
AFM images of DOPC:lSM:Chol-based SPB. DOPC:lSM:Chol (2:1:1) + 30% lCer (**A**), + 30% nCer (**B**), + 15% lCer and 15% nCer (**C**). Temperature was 23 ± 1 °C.

DOPC:lSM:Chol (2:1:1) samples containing additional 30 mol% Cer (either lCer, nCer or a 15 mol% + 15 mol% mixture of each), were prepared. Confocal microscopy imaging of GUV (Fig. 2a–c) showed segregated gel phases by RhoPE depletion, except for the 30 mol% lCer mixture (Fig. 2a), as domains were not detected in that sample. Figure 2c showed two different segregated phases detected by different degrees of fluorophore partition (see zoom-in in Fig. 3a,b). In AFM experiments of SPBs (Fig. 4) it was checked that, as initially expected, the three Cer-containing mixtures presented gel domains with a significantly increased nanomechanical resistance, the most rigid domains being those containing lCer (Table 1). Domain shapes in Fig. 4a were of particular interest as they did not present the classical rounded or flower-like boundaries reported for most Cer-domains in SPBs^13,54^ or GUV^32^, rather fern-like or needle-like domains were seen instead. In Fig. 4c two different types of segregated phases were observed, which exhibited different nanomechanical resistances (Table 1). This was in agreement with Figs. 2c and 3b, as there are three phases present (one fully stained, another one very faintly stained, and the third one completely fluorescence-depleted), which could be explained respectively as a fluid phase (lowest nanomechanical resistance of the three, as shown in Table 1), a gel phase enriched in nCer (intermediate nanomechanical resistance) and a gel phase rich in lCer (highest stiffness).

**Table 1:** Bilayer nanomechanical resistance of the different SPB under study.

		Breakthrough force (nN)	
		Continuous phase	Segregated domains
DOPC:lSM:Chol	+ lCer	7.0 ± 2.3	54.5 ± 12.0
	+ nCer	4.8 ± 2.1	15.2 ± 1.2
	+ lCer + nCer	2.3 ± 0.9	10.38 ± 1.57
			16.38 ± 1.36
DOPC:nSM:Chol	+ lCer	4.1 ± 1.7	23.3 ± 6.9
	+ nCer	1.5 ± 0.6	6.2 ± 2.4
	+ lCer + nCer	2.0 ± 0.7	9.6 ± 1.9
DOPC:lSM:nSM:Chol	+ lCer	3.7 ± 1.9	31.9 ± 4.5
	+ nCer	2.3 ± 0.8	7.2 ± 1.7
	+ lCer + nCer	2.6 ± 1.9	11.3 ± 2.2

Data expressed as mean ± SD, n = 300–1,000.

In order to check whether phase separation observed in GUV could be the result of metastability, GUV prepared as in Fig. 2c, with DOPC:lSM:Chol (2:1:1) + 15% lCer + 15% nCer, were subjected to a slow heating–cooling process (5 °C/5 min; 25–80–25 °C). The results (not shown) failed to detect any difference in phase separation before and after the heating–cooling process, indicating that the observed phase separation was not the result of poorly equilibrated, metastable samples.

**Figure 5:**
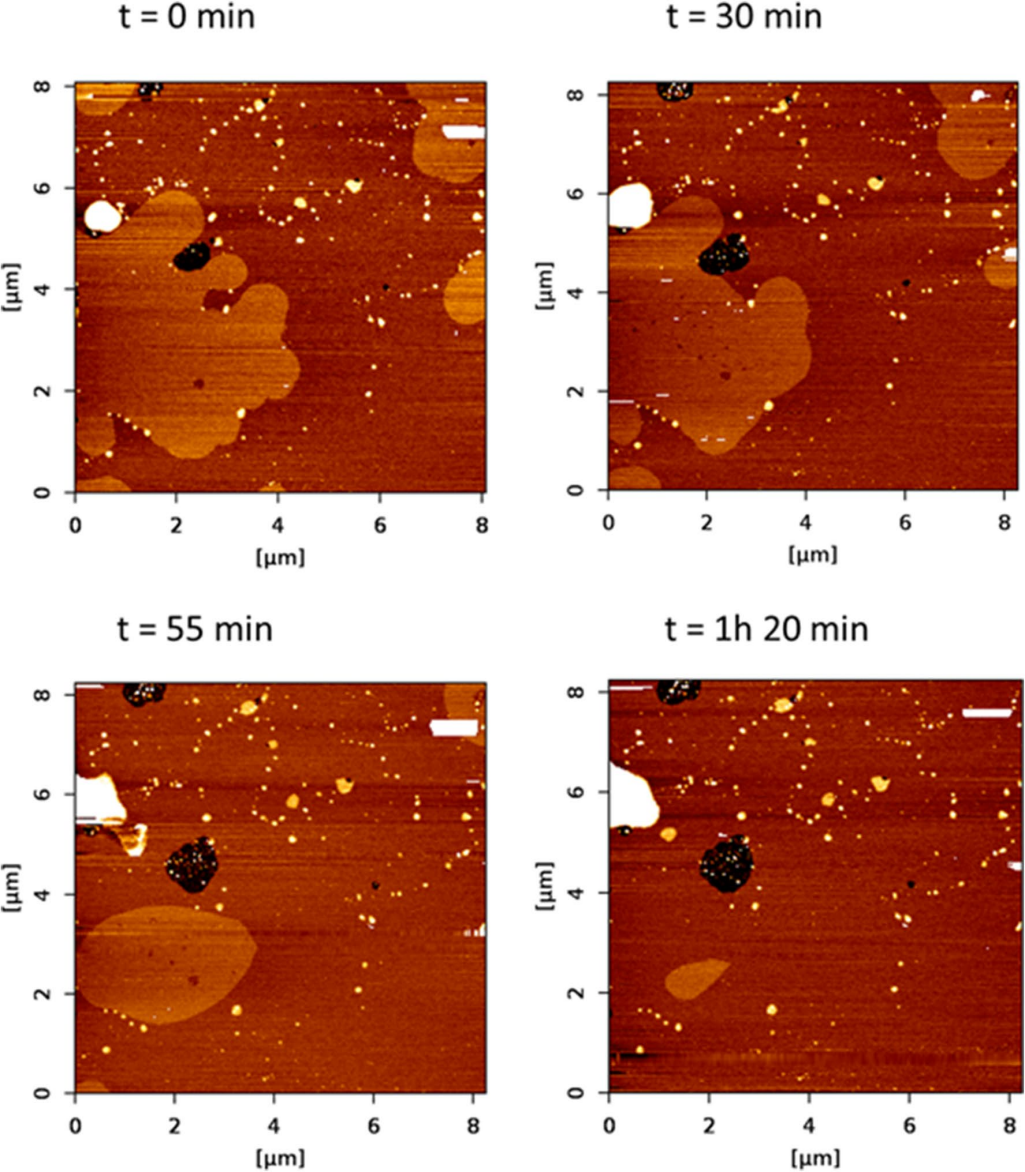
Bilayer dynamism in C24:0 + C24:1 sphingolipid samples containing Cer: a representative example. AFM images of SPBs of DOPC:lSM:Chol:nCer (from the same SPB as depicted in Fig. 4b). As time progressed, with T kept constant at 23 °C as usual, the size of the segregated gel domains was progressively reduced, while the size of non-lipid coated areas (black zone in the center-left part of the image) increased. The size of the aggregated multiple bilayers (white coloured areas in the upper-left part of the image) also became larger.

**Figure 6:**
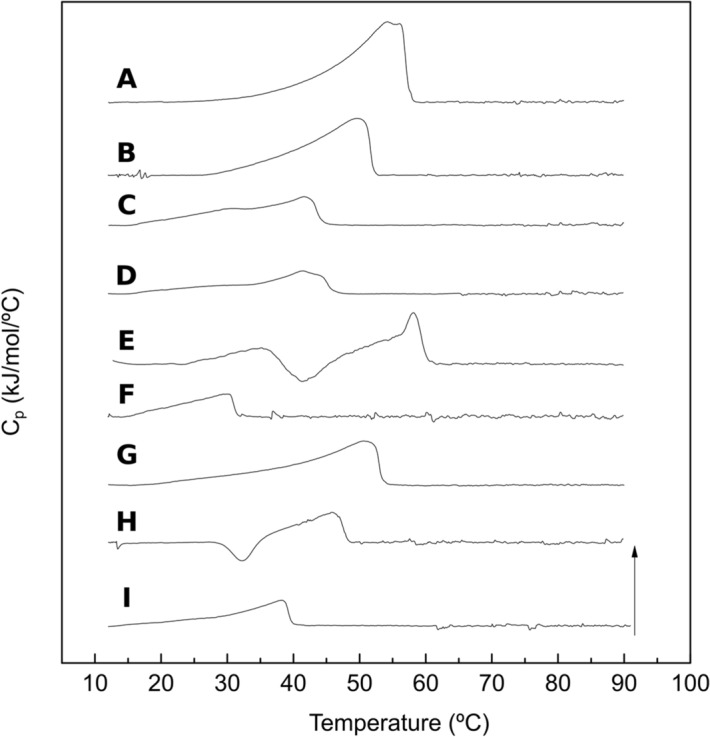
DSC thermograms of lipid mixtures dispersed in excess water. DSC thermograms. DOPC:lSM:Chol (2:1:1) + 30% lCer (**A**), + 15% lCer and 15% nCer (**B**), + 30% nCer (**C**); DOPC:nSM:Chol (2:1:1) + 30% lCer (**D**), + 15% lCer & 15% nCer (**E**), + 30% nCer (**F**); DOPC:lSM:nSM:Chol (2:0.5:0.5:1) + 30% lCer (**G**), + 15% lCer and 15% nCer (**H**), + 30% nCer (**I**). Arrow represents 8 kJ/mol °C**.**

Domains in SPBs showed a tendency to disappear gradually over time (in a process that was completed after about 2 h) in DOPC:lSM:Chol:nCer (Fig. 4b) and DOPC:lSM:Chol:nCer:lCer (Fig. 4c) but not in the DOPC:lSM:Chol:lCer mixture (Fig. 4a) as the latter remained in equilibrium. An example of the topographical monitorization of the process can be found in Fig. 5. In addition, the nanomechanical resistance values detected for the domains did not change during the process, nor did the values for the continuous fluid phases. This will be further assessed in the “Discussion” section. Furthermore, DSC results (Fig. 6a–c, Table 2) indicate that the 30 mol% lCer mixture exhibited the highest T_m_ of all samples in the present study (Fig. 6a, Table 2), while the presence of nCer made T_m_ increase to a lesser extent (Fig. 6b,c). In summary, both ceramides were able to form gel phase domains, and the presence of lCer increased rigidity of the sample more than nCer, as expected from the N-acyl unsaturation in the latter.

**Table 2:** Thermodynamic parameters of the gel-fluid transitions, as obtained from DSC experiments.

		∆H (kJ/mol °C)	T_m_ (°C)	T_1/2_ (°C)
DOPC:lSM:Chol	+ lCer	87.6 ± 12.6	54.4 ± 0.2	10.0 ± 0.7
	+ lCer + nCer	63.2 ± 3.4	39.5 ± 0.3	11.2 ± 1.1
	+ nCer	35.8 ± 1.7	41.5 ± 0.4	14.1 ± 1.2
DOPC:nSM:Chol	+ lCer	28.4 ± 1.4	41.7 ± 0.4	9.4 ± 0.1
	+ lCer + nCer	33.0 ± 4.5	55.2 ± 4.6	39.5 ± 3.4
	+ nCer	17.9 ± 1.2	30.9 ± 1.2	8.0 ± 1.0
DOPC:lSM:nSM:Chol	+ lCer	59.9 ± 1.4	51.2 ± 0.5	10.5 ± 1.0
	+ lCer + nCer	12.8 ± 4.1	47.7 ± 2.4	8.0 ± 0.5
	+ nCer	23.4 ± 0.9	38.7 ± 0.7	7.7 ± 0.1

Average values ± SD (n = 3). For simplicity, in the case of multiple transition events (Fig. 6e,h) the data presented here correspond to the integrated absolute values of the total transitions.

### nSM-based samples

**Figure 7:**
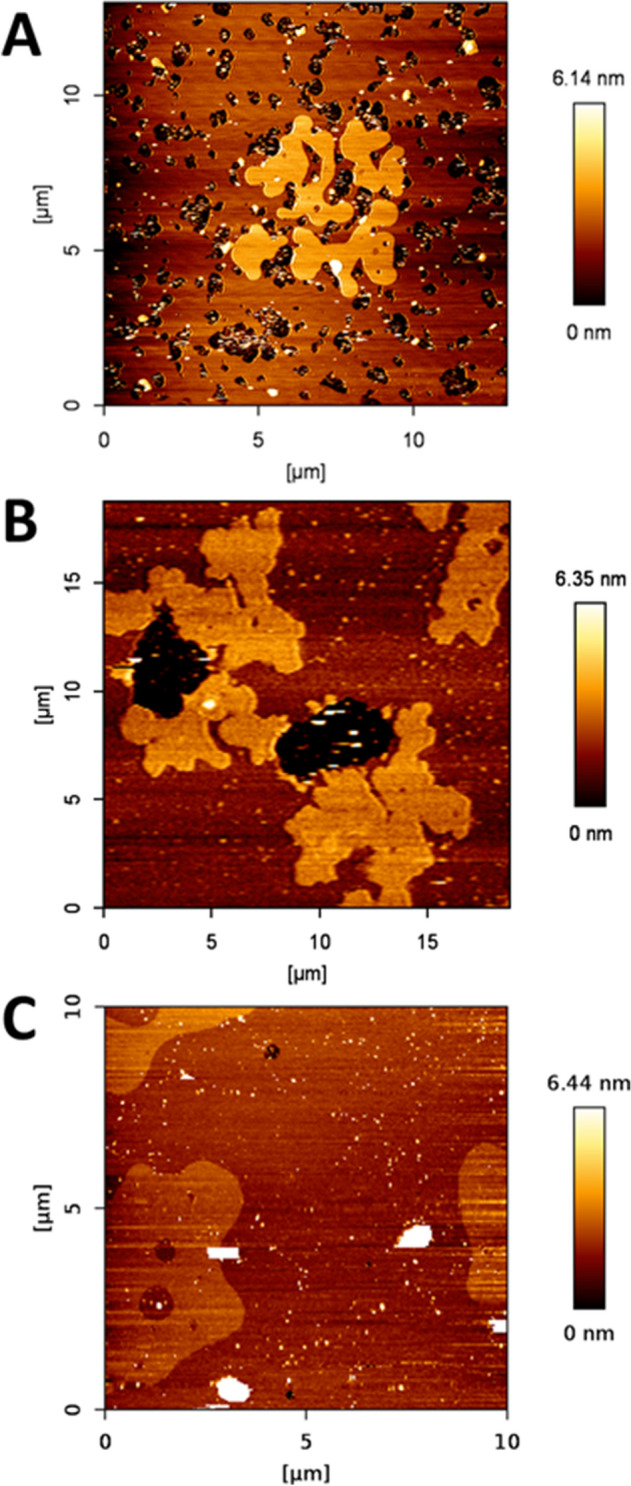
AFM images of DOPC:nSM:Chol-based SPB. DOPC:nSM:Chol (2:1:1) + 30% lCer (**A**), + 30% nCer (**B**), + 15% lCer and 15% nCer (**C**). Temperature was 23 ± 1 °C.

Following the same approach as with lSM, experiments were performed with DOPC:nSM:Chol (2:1:1) + 30% Cer (lCer, or nCer. or a nCer/lCer equimolar mixture). In this case, segregated domains could be seen in GUV prepared with each of the three samples (Fig. 2d–f), in the form of areas with little or no fluorescence. Figure 2f also showed a second, completely florescence-depleted, segregated phase within the predominant segregated phase, although it was rather difficult to distinguish. A zoom-in of the GUV is shown in Fig. 3c,d to demonstrate this point. In AFM experiments, the segregated gel phases were detected as well (Fig. 7) with the usual rounded interphase boundaries, but Fig. 7c showed only one type of segregated domain, and not two, as seen in the fluorescence picture 2F. It cannot be ruled out that two gel phases existed in SPB in the present case, yet AFM was not able to properly resolve them in either topography (Fig. 7c) or force spectroscopy (Table 1) modes, but noting the tiny difference in RhoPE partition between them (Fig. 2f), it could be assumed that these two were of a very similar lipid composition, thus endowed with equally similar properties. In terms of nanomechanical resistance (Table 1), a clear decrease was patent going from lSM to nSM and from lCer to nCer across the samples, breakthrough forces for nSM-based mixtures being reduced by almost one half with respect to the values detected in the lSM-based set. Moreover, the decrease was also detected in the nanomechanical resistance values for both the rigid and the fluid phases, showing that the substitution of nSM for lSM affected the whole membrane, and not only the more rigid segregated phases. In addition, two of the three samples (the exception being DOPC:nSM:Chol:nCer) showed the bilayer dynamism mentioned above, following the trend shown in Fig. 5: gel phases tended to decrease in size and finally disappeared over time, with constant nanomechanical resistance values through the process (see “Discussion” section). An additional example of bilayer dynamism is shown in Supp. Fig. S2 for the bilayer in Fig. 7c.

DSC experiments (Fig. 6d–f, Table 2) were in agreement with the previous data, as the T_m_ decreased when an increased amount of nCer was present. The nSM-nCer system (i.e. without the presence of any C24:0 sphingolipid), which had been previously reported in García-Arribas et al.^37^, exhibits the lowest T_m_ of all the data sets under study. The DOPC:nSM:Chol:lCer:nCer thermogram (Fig. 6e) exhibited a complex transition with an exothermic region, and the measured T_m_ was in this sample higher than expected, perhaps due to software limitations to precisely identify T_m_ in such complex thermograms. The presence of exothermic signals in this and other samples in the study will be further commented on in the “Discussion” section. Exothermic signals were a reproducible feature of these samples, and their presence was not influenced by sample preparation procedures or long equilibration periods. In summary, the substitution of lSM by nSM did not override domain formation, but T_m_ values decreased greatly and there was a general reduction in bilayer stiffness for every phase present.

### Samples containing both lSM and nSM

**Figure 8:**
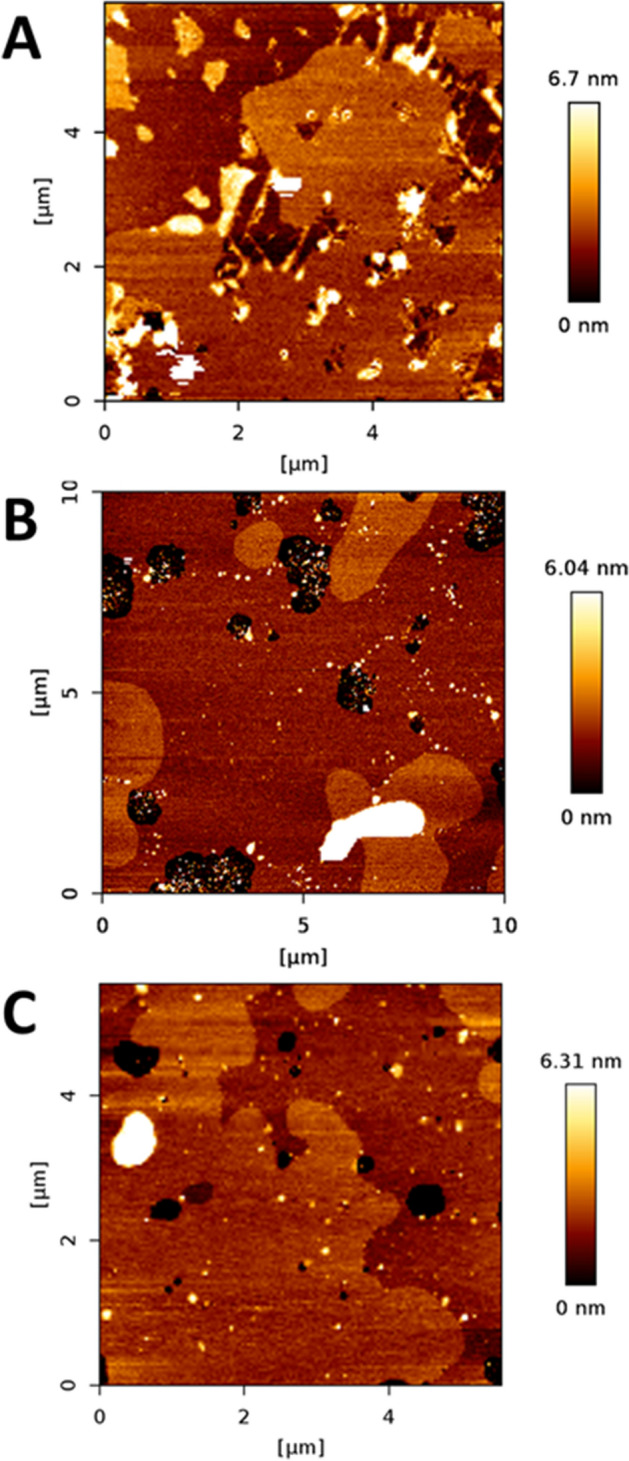
AFM images of DOPC:lSM:nSM:Chol-based SPB. DOPC:lSM:nSM:Chol (2:0.5:0.5:1) + 30% lCer (**A**), + 30% nCer (**B**), + 15% lCer and 15% nCer (**C**). Temperature was 23 ± 1 °C.

The case of DOPC:lSM:nSM:Chol (2:0.5:0.5:1) + 30% Cer was the most complex sample set of our study. Again, and as expected, segregated gel phases were observed using confocal microscopy (Fig. 2g for lCer, Fig. 2h for nCer and Fig. 2i for the mixture of both ceramides). As in the previous data sets, the two-Cer sample exhibited two segregated phases in GUV (Fig. 2i), with three levels of fluorescent staining (one fully stained phase, a second, slightly stained phase, and a third one completely non-stained). However, the partially unstained phase was hard to distinguish from the completely non-stained one, as in Fig. 2f. The zoom-in panel in Fig. 3e,f clarifies this phase separation. AFM imaging showed segregated phases for each sample in SPBs (Fig. 8), these domains also had a tendency to disappear over time, while keeping invariant their nanomechanical resistance values, as in the previous data sets. In Fig. 8a a tendency of domains to form linear structures (center and top-right hand quarter of the image, somehow similar to those in Fig. 4a) at the domain boundaries was also detected, which could be caused by the lSM-lCer interaction. The two-Cer sample presented a single segregated phase identifiable by topography (Fig. 8c) or force spectroscopy (Table 1), which was also the case for the nSM-based two-Cer sample (Fig. 7c). DSC results (Fig. 6g–i, Table 2 followed the same trend as in the previous data sets, lCer increased T_m_ and nCer lowered it. The two-Cer sample (Fig. 6h) showed a rather complex behavior, including an exothermic component before the main endothermic transition. Exotherms were also present in the two-Cer nSM-based sample (Fig. 6e) and similarities between these two data sets and putative conclusions will be commented on in the “Discussion” section.

Regarding the relative height data of the segregated domains (Table S1), while the tendency resembles the one of force spectroscopy results, there are some interesting details. The nSM-nCer system exhibits a large relative height, as previously reported^37^, comparable to the nSM-lCer system, but suffers a drastic decrease when both ceramides are combined, suggesting some different lipid rearrangement. According to the force spectroscopy data, this phase would have an intermediate nanomechanical resistance between both nSM-lCer and nSM-nCer systems, but much closer to the nSM-nCer values. Note also that the presence of nCer does not cause a comparable sharp increase in relative height in any sample other than the nSM-nCer system, which indicates that the lipid arrangement of this segregated phase is highly susceptible to the presence of 24:0 SM and Cer species, but not to 16:0 sphingolipids^37^. This speaks in favor of the capacity of 24:1 and 24:0 sphingolipids to accommodate each other in a more efficient fashion than their 16:0 counterparts.

## Discussion

The above results reveal several aspects worthy of a separate discussion. In terms of nanomechanical resistance, and of T_m_ calculated by DSC experiments, the results were fairly consistent, as lSM-based samples exhibited transitions at higher T_m_ and were more resistant to AFM probe indentation than those based on nSM. Cer also had a comparable impact on both data sets, as lCer was a much better stiffening agent than nCer. These conclusions are in agreement with those by García-Arribas et al.^37^, who used pSM and pCer instead of lSM and lCer, respectively. However, that study did not encounter some of the issues reported here, such as (i) the absence of domains in GUV (Fig. 2a) where SPB did show them (Fig. 4a), (ii) the presence of exothermic signals in thermograms (Fig. 6e,h), or (iii) the observation of bilayer dynamism/instability (Fig. 5, S1, S2).

With respect to the AFM–DSC correlations, an interesting detail of SPB topography is that the phase arrangement in Fig. 4c resembles those of DOPC:pSM:Chol:pCer:nCer^37^, with one of the segregated phases embedded into another segregated phase. However, in terms of nanomechanical resistance the profiles of both samples differ greatly as the ‘intermediate’ phase exhibits much higher stiffness in the present case (10.38 vs. 3.7 nN^37^). This points to lCer as a better rigidifying agent than pCer in the presence of other unsaturated Cer. This is further supported by DSC results which show that lCer, in the presence of lSM (and absence of nSM), has an increased efficiency for membrane stiffening when compared to, for instance, pSM and pCer in the same quaternary system (54.4 °C vs. 48.5 °C^37^). However, the same report by García-Arribas et al.^37^ included the force spectroscopy data for the DOPC:pSM:Chol:pCer system, which were similar to those reported here for DOPC:lSM:Chol:lCer both fluid and gel phases. This is interesting because T_m_ usually correlates almost perfectly with force spectroscopy data of gel phases (as gel phases are more resistant to AFM indentation, and they typically show higher T_m_), but this was not the case in the latter sample (higher T_m_ with equivalent breakthrough force).

Regarding the notable exception of Fig. 2a not showing domains as found in equivalent samples, in this and previous studies, and considering the DSC thermogram in Fig. 6a, an explanation would be that these GUV are, at least macroscopically, in the gel state. The situation could be related to the unusual needle-shaped domain morphology in Fig. 4a. GUV of POPC:lCer (70:15) reported by Pinto et al.^32^ showed that although there was not a clear presence of domains, some tubular structures were present, which, in a planar shape, would resemble the needle-like domains of our SPB in Fig. 4a. Thus, we cannot discard the possibility of the domains not being able to properly assemble in the electroformation process of GUV for this sample, due to the presence of these tubular structures, while the SPBs are extended smoothly from bath-sonicated SUV at a T above the T_m_ of the sample.

Comparing the results for DOPC:nSM:Chol:lCer:nCer and DOPC:lSM:nSM:Chol:lCer:nCer is also relevant and can provide further insights on the complexity of these systems. As mentioned under Results the above two mixtures exhibit some particular similarities: (i) GUV show the same number of phases with almost the same relative degree of staining (Figs. 2f,i, 3d,f), (ii) AFM detects a single segregated phase (Figs. 7c, 8c), (iii) nanomechanical resistance results for the domains (Table 1) indicate that their values are close to each other (although with a statistically significant difference), and (iv) DSC results (Fig. 6, Table 2) show that the T_m_ for Fig. 6h is lower than Fig. 6e (Table 2) but, interestingly, both thermograms present exotherms and are the only ones in our study to do so. These results point to the presence of lSM in a nSM-containing sample as a molecule that increases rigidity but, apparently, does not affect the distribution of both Cers, the latter being seemingly governed by nSM.

The presence of exotherms, which appear only in the aforementioned two samples (Fig. 6e,h), could be interpreted as the reorganization of quasi-solid aggregates (Cer is able to behave as a solid^55^ and undergo some molecular rearrangements^56^) which reportedly exhibit exotherms before endothermic transitions in DSC experiments for other highly hydrophobic non-lipid molecules such as curcumin^57^. However, these putative aggregates would only appear in the nSM-containing two-Cer samples, regardless of the presence or absence of lSM.

As for the causes of bilayer dynamism/instability, one outstanding candidate would be the mica support, although experiments to prove it are not easy to design in practice. For the Cer-less samples we initially conceived the instability as the combined effect of T and time, perhaps related to some non-thermotropic transition at room temperature. However, the experiments in Supp. Fig S1 indicated that T was not a determinant factor in the presence or absence of domains, and further studies with Cer showed that the instability was time dependent. The experiment in Supp. Fig S1 showed that T could affect the size of domains, but not their presence nor the capacity of AFM to detect them. If domains required T > 23 °C to be visible, they should be more prominent and grow at 27 °C, but this was not the case, in fact they decreased in size, and if they required T < 23 °C they should disappear completely from the image at 27 °C, but this was not the case either, they were still clearly visible. These observations led us to the conclusion that T was not the determining factor in the process of domain rearrangements.

All SPBs under study showed some degree of bilayer dynamism except for DOPC:lSM:Chol:lCer and DOPC:nSM:Chol:nCer. This led us to the conclusion that bilayer dynamism is not only enhanced by the mica support (as it does not happen in GUV) but also by the joint presence of 24:0 and 24:1 sphingolipids, SM and/or Cer, in the same bilayer. For the Cer-containing samples (except those two samples in equilibrium mentioned above), domain disappearance also caused a reduction in bilayer extension of the SPBs, as the non-coated mica areas grew larger and, at the same time, the appearance of aggregates of greater thickness and increasing size was detected (this did not happen in the Cer-less quaternary sample, as demonstrated in Supp. Fig. S1). All these features occur in the examples shown in Fig. 5 and Supp. Fig. S2, the latter also showing the final disappearance of the domains. The fact that the nanomechanical resistance of the different lipid phases present does not change during the dynamic process probably points to the lipid:lipid stoichiometry not being altered within the domains, which would mean that the domains are being spontaneously ‘disassembled’ by, perhaps, some molecular mismatch effect on the interphase boundary that affects their size^58^. An additional hypothesis would be that Chol would be gradually released from complexes with SM, thus progressively lowering line tension and inducing a microscopic-to-nanoscopic transition for the domains^59^. The possibility that the micron-size domains are eventually and spontaneously rearranging themselves as nanometric clusters cannot be discarded, as these clusters would be below the resolution of the AFM tip^60^, and probably they would not exhibit biophysical properties different from those of the continuous phase^52^. The (yet unproven) phenomenon would be one of changing domain sizes but not a phase transition^52^.

It may be relevant in this context the observation that in the DSC experiments, when the samples are heated and cooled repeatedly, and particularly for the more complex samples, of 5 and 6 components, a stable, reproducible thermogram is not seen before the fourth or fifth heating–cooling cycle (not shown). This is probably reflecting the same phenomenon of unstable bilayers observed by AFM, and it would speak against a strict dependence of mica for the instability in the latter images.

The idea that the instability could be triggered by the scanning process of the sample, which would explain the constant observation of sizable domains at t = 0, was considered, but bilayer dynamism was not only limited to the scanned areas: domains also disappeared in non-previously-scanned zones of the SPBs. Thus, we can only conclude that while SPB dynamism also appeared in the absence of Cer (Supp. Fig. S1), the effect was different when Cer was present (Fig. 5 and Supp. Fig. S2) and that both effects require the presence of 24:0 and 24:1 sphingolipids in the same SPB.

Regarding the miscibility and the capacity of lCer to interact with other lipids in multicomponent bilayers (and particularly with Chol), lCer seems to behave much like pCer, albeit with some clear differences. On one hand, lCer is more miscible in cases as shown in Fig. 5b: pCer in the equivalent case gave rise to two segregated domains^37^, suggesting that lCer is easier to accommodate with nCer in the gel phase in the presence of nSM. On the other hand, lCer presents less miscibility in cases as in Fig. 4c, in which both segregated domains are Cer-enriched gel phases, while in the case of pSM and pCer there was one gel phase with both Cer and one liquid-ordered phase without Cer^37^. The apparent conclusion here points to lCer being more miscible than pCer with nCer in the presence of nSM, and less miscible with nCer in the presence of lSM (instead of pSM). In summary, lCer is easier to accommodate in a phase with a different N-acyl SM-Cer system, while it seems to interact more avidly with its own N-acyl SM (lSM), hampering the lSM-lCer system capacity to accommodate other species of N-acyl Cer. This is compatible with our previously stated conclusion about the nSM as a ‘governing’ factor for Cer distribution. Related to this conclusion, it is reasonable to consider that lCer (and lSM) could have a much higher tendency to interdigitation than their C16:0 counterparts^61^, as the capacity of lSM to present interdigitation has been reported^35^. This points to lCer as a very suitable Cer to form stiff gel domains that could also accommodate a significant amount of other lipids such as Chol, as pCer is able to do under specific conditions of high Chol and pCer concentrations^40,47^, or in a lipid environment deprived of a fluid phase^46^. In summary, while pCer and Chol have a strong tendency to displace each other from the same interaction sites^62–64^, lCer (and lSM) could offer a solution for cells to fine tune the biophysical properties of their membranes, particularly in the presence of a wide variety of different N-acyl sphingolipids, which is the situation in living cell membranes.

Cholesterol exerts a series of effects (not all of them well understood) on the dynamics of lipid bilayers. In the context of the present study, at least the following should be taken into account: (i) the well-known Chol property of blurring the gel-fluid phase transition of saturated phospholipids, including SM^65,66^, (ii) the competition of Chol and Cer in binding SM^62,67^, and (iii) the capacity of Chol to lower line tension, thus facilitating the conversion of microdomains into nanodomains^59^. Investigating which of these effects predominates in the individual mixtures will be the object of future research.

In conclusion, our study of C24:0 and C24:1 sphingolipids and their influence on multicomponent cholesterol-containing lipid bilayers is a good example of the impact of sphingomyelins and ceramides on the biophysical properties of membranes. C24:0 SM and C24:0 Cer act in a way that could resemble their C16:0 counterparts in general (gel phase segregation, membrane stiffening, increase in transition temperatures), but with some peculiarities that could affect the living cell condition. For instance, for the DOPC:lSM:Chol:lCer system an unusual non-rounded shape for the gel domains is detected. Moreover, combination of C24:0 and C24:1 sphingolipids generates: (i) bilayer instability in supported planar bilayers that affects gel phase domain appearance/disappearance, (ii) comparison between GUV–AFM experiments reveal unusual discrepancies, and (iii) exotherms appear in some thermotropic transitions. Moreover, C24:0 SM appears as having a lesser impact on the distribution of a combination of both Cer, while C24:1 SM seems to be the key lipid in that regard. In addition, C24:0 Cer seems to be easier than C16:0 Cer to accommodate in the presence of C24:1 sphingolipids. These results could provide a molecular basis for understanding the consequences of sphingomyelinase activation in the cell plasma membrane, at the onset of the sphingolipid signalling pathway.

## Materials and methods

### Chemicals

1,2-Dioleoyl-*sn*-glycero-3-phosphocholine (DOPC), N-lignoceroyl-D-erythro-sphingosylphosphorylcholine (24:0 SM, lSM), N-nervonoyl-D-erythro-sphingosyl-phosphorylcholine (24:1 SM, nSM), N-lignoceroyl-D-erythro-sphingosine (24:0 Cer, lCer), N-nervonoyl-D-erythro-sphingosine (24:1 Cer, nCer), cholesterol (Chol), and the lipophilic fluorescent probe 1,2-dioleoyl-*sn*-glycero-3-phosphoethanolamine-N-(lissamine rhodamine B sulfonyl) (Rho-PE) were purchased from Avanti Polar Lipids (Alabaster, AL). DiI (1,1′-Dioctadecyl-3,3,3′,3′-Tetramethylindocarbocyanine Perchlorate ('DiI'; DiIC18(3))) was supplied by Thermo Fisher (Waltham, MA, USA). Methanol and chloroform were from Fisher (Suwanee, GA). Buffer solution, unless otherwise stated, was 20 mM PIPES, 1 mM EDTA, 150 mM NaCl, pH 7.4. All salts and organic solvents were of analytical grade.

### Liposome preparation

All lipid mixtures are given as mole ratios. Lipid vesicles were prepared as described in a previous report^37^, by mixing the desired lipids dissolved in chloroform/methanol (2:1, v/v) and drying the solvent under a stream of nitrogen. The lipid film was kept under high vacuum for 90 min to ensure the removal of undesired organic solvent. Multilamellar vesicles (MLV) were formed by hydrating the lipid film with the buffer solution at 90 °C, helping the dispersion with a glass rod. The samples were finally sonicated for 10 min in a bath sonicator at the same temperature, to facilitate homogenization.

### Confocal microscopy of giant unilamellar vesicles (GUVs)

GUVs are prepared as described previously, using the electroformation method developed by Angelova and Dimitrov^68,69^. Lipid stock solutions were prepared in 2:1 (v/v) chloroform/methanol and appropriate volumes of each preparation were mixed. Labelling was carried out by pre-mixing the desired fluorescent probe (Rho-PE) with the lipids in organic solvent. Fluorescent probe concentration was 0.4 mol% Rho-DOPE. No bleaching of the probe, due to oxidation or otherwise^70^, could be detected under our conditions. The use of a low probe concentration, and the unsaturation in the acyl chain ensured the absence of unwanted probe effects^71,72^ such as distortion of phase separation and changes in bending modulus of the membrane^73,74^. Selected samples were stained wth DiI (1,1′-Dioctadecyl-3,3,3′,3′-Tetramethylindocarbocyanine Perchlorate ('DiI'; DiIC18(3))), the results were essentially identical to those obtained with Rho-DOPE (data not shown). As an additional test that probe oxidation was not perturbing the GUV results, a representative complex sample, DOPC/lSM/Chol (2:1:1 mol ratio) + 15 mol% nCer + 15 mol% lCer was prepared with RhoPE but, instead of GUV, SUV were prepared and extended onto a mica surface using the vesicle adsorption method described below for supported planar bilayer formation. Fluorescence images were taken with a Leica epifluorescence microscope (DMI 4000B) in which three phases were distinguishable. AFM topographical images of the same preparation showed as well three phases (images not shown). This control experiment confirms that the capacity of Rho-PE to partition between different phases is not affected by the GUV electroformation method. The problem of oxidation is irrelevant for AFM, yet three phases are observed by AFM as well as with Rho-PE.

GUV preparation was as follows. The samples were deposited onto the surface of platinum (Pt) wires attached to specially designed polytetrafluoroethylene (PTFE)-made cylindrical units that were placed under vacuum for 2 h to completely remove the organic solvent. The sample was covered to avoid light exposure. Then, the units were fitted into specific holes within a specially designed chamber (Industrias Técnicas ITC, Bilbao, Spain), to which a glass cover slip had been previously attached with epoxy glue. Once fitted, the platinum wires stayed in direct contact with the glass cover slip. The chamber was then equilibrated at the desired temperature by an incorporated water bath. 400 µL 10 mg/mL sucrose, prepared with high-purity water (SuperQ, Millipore, Billerica, MA) and heated at 90 °C, were added until the solution covered the Pt wires. The chambers were stopped with tightly fitting caps, and connected to a TG330 function generator (Thurlby Thandar Instruments, Huntingdon, UK). The alternating current field was applied with a frequency of 10 Hz and an amplitude of 940 mVrms for 300 min. The temperatures used for GUV formation were above the gel-to-liquid phase transition in all cases (≈90 °C). The generator and the water bath were switched off, and vesicles were left to equilibrate at room temperature for 60 min. A slow cooling was needed to observe large enough domains by fluorescence microscopy^75^. After GUV formation, the chamber was placed onto an inverted confocal fluorescence microscope (Nikon D-ECLIPSE C1, Nikon, Melville, NY), and examined at 23 ± 1 °C. The excitation wavelength was 561 nm. Emission was retrieved between 573 and 613 nm. Image treatment and quantitation were performed using the software EZ-C1 3.20 (Nikon). No difference in domain size, formation, or distribution was detected in the GUV during the observation period or after laser exposure. Note that, in general, GUV composition does not necessarily reflect in an exact way the composition of the lipid mixture used in their preparation. With the samples used in the present study this could be important, Cer being difficult to hydrate. However, the parallel use of calorimetry and AFM with the same samples provided coherent results, supporting the assumption that the effective GUV composition was at least close to the nominal one.

The GUV experiments were performed on at least three independent preparations for each sample. Six independent preparations were made of DOPC:lSM:Chol (2:1:1) + 30% lCer samples, due to the absence of non-stained phases in these samples. For each sample approximately fifty GUV were screened, and no pattern heterogeneity was observed, thus the vesicles shown in Figs. 2–3 are representative of each sample.

### Supported planar bilayer (SPB) formation

SPB were prepared on high V-2 quality scratch-free mica substrates (Asheville-Schoonmaker Mica Co., Newport News, VA) previously attached to round 24 mm glass coverslips by the use of a two-component optical epoxy resin (EPO-TEK 301-2FL, Epoxy Technology Inc., Billerica, MA). SPB were obtained following the vesicle adsorption method^76,77^. MLV, prepared as described above, were introduced in a FB-15049 (Fisher Scientific Inc., Waltham, MA) bath sonicator and kept at 70 °C for 1 h. In this way a proportion of small unilamellar vesicles (SUVs) were generated. Thereafter, 120 µL assay buffer containing 3 mM CaCl_2_ were added onto previously prepared 1.2 cm^2^ freshly cleaved mica substrate mounted onto a BioCell coverslip-based liquid cell for atomic force microscopy (AFM) measurements (JPK Instruments, Berlin, Germany). 60 µL sonicated vesicles were then added on top of the mica. Divalent cations such as Ca^2+^ or Mg^2+^ have been described as enhancers of the vesicle adsorption process onto mica substrates^78^. Final lipid concentration was 150 μM. Vesicles were left to adsorb and extend for 30 min keeping the sample temperature at 60 °C. In order to avoid sample evaporation and ion concentration, after the first 5 min the buffer was constantly exchanged with assay buffer without CaCl_2_ at 60 °C for the remaining time. Additional 30 min were left for the samples to equilibrate at room temperature, discarding the non-adsorbed vesicles by washing the samples 10 times with assay buffer without CaCl_2_, in order to remove remaining Ca^2+^ cations, which are reported to drastically affect the breakthrough force (F_b_) results of lipid bilayer nanoindentation processes^45^_._ The efficiency of repeated rinsing to obtain proper and clean supported lipid bilayers has been reported^79^. This extension and cleaning procedure allowed the formation of bilayers that did not cover the entire substrate surface. The presence of lipid-depleted areas helped with the quantification of bilayer thicknesses and the performance of proper controls for force-spectroscopy measurements. Planar bilayers were then left to equilibrate at room temperature for 1 h prior to measurements in order to avoid the presence of possible artifacts as segregated domains appear at high temperatures (over the T_m_)^80^ and could still be present at lower temperatures if the cooling process was too fast (> 1 °C/min)^78^. Finally, the BioCell was set to 23 °C to start the AFM measurements.

### Differential scanning calorimetry (DSC)

The measurements were performed in a VP-DSC high-sensitivity scanning microcalorimeter (MicroCal, Northampton, MA). Sample preparation (3 independent preparations per sample) and data analysis were performed as described in a previous report^37^. MLV to a final concentration of 1 mM (2 mM in some cases) were prepared as described above with a slightly different hydration step: instead of adding the buffer solution in a single step, increasing amounts of the solution were added, helping the dispersion by stirring with a glass rod. Then the vesicles were homogenized by forcing the sample 50–100 times between two syringes through a narrow tube (0.5 mm internal diameter, 10 cm long) at a temperature above the transition temperature of the lipid mixture. Before loading the MLV sample into the appropriate cell both lipid and buffer solutions were degassed. 0.5 mL of suspension containing 1 mM total lipid concentration (2 mM for samples E and H in Fig. 6, to improve signal-to-noise ratio) were loaded into the calorimeter, performing 8 heating scans at a 45 °C/h rate, between 10 and 90 °C for all samples. Cooling scans were also recorded at the same 45 °C/h rate for selected samples, but no additional information could be retrieved (data not shown). The software Origin 7.0 (MicroCal), provided with the calorimeter, was used to determine the different thermodynamic parameters from the scans. The procedure for baseline subtraction in DSC experiments was as follows. First, a buffer scan was measured in the microcalorimeter to remove the buffer effect from the thermal transition of the lipids of interest. Then, the sample was measured and, after measurement, the software Origin version 7.0 (https://www.originlab.com/) provided with the microcalorimeter automatically subtracted the baseline from the sample scan. Moreover, a cubic adjustment was performed to obtain a flat line before and after the transition temperature. Finally, the phospholipid concentration was determined using a phosphorus assay and the thermogram was normalized to the measured concentration. Further information on calorimetric studies of ceramide-containing samples can be found elsewhere^81^.

### AFM imaging

Planar bilayer topography was performed in an UltraSpeed AFM (JPK Instruments, Berlin, Germany) using the ‘QI Mode’, an imaging mode that performs force curves simultaneously at a low force (< 1 nN). For proper measurements the AFM was coupled to a Leica microscope and mounted onto an anti-vibration table and inside an acoustic enclosure (JPK Instruments), in a similar setup and experimental approach as described previously^37^. The BioCell liquid sample holder (JPK Instruments) was used in order to control the assay temperature at 23 ± 1 °C. V-shaped MLCT Si_3_N_4_ cantilevers (Bruker, Billerica, MA) with nominal spring constants of 0.1 or 0.5 N/m were used for bilayer imaging, obtaining 256 × 256 pixel images though ‘QI Mode’. Images were line-fitted using the JPK Data Processing software, version 6.1.142 (https://customers.jpk.com/). Images are representative of the samples after 3 independent experiments.

### Force spectroscopy

Force spectroscopy experiments and data analysis were performed as described in a previous report^37^. V-shaped MLCT Si_3_N_4_ cantilevers (Bruker, Billerica, MA) with nominal spring constants of 0.1 or 0.5 N/m were individually calibrated in a lipid-free mica substrate in assay buffer using the thermal noise method. After proper bilayer area localization by AFM topography, force spectroscopy was performed at a speed of 1 μm/sec in no less than 500 × 500 nm bilayer areas in the form of 10 × 10 or 12 × 12 grids. Force steps were determined for each of the indentation curves as reproducible jumps within the extended traces. The resulting histograms were generated from at least 3 independent sample preparations with at least 3 independently calibrated cantilevers (n = 300–1,000). Control indentations were always performed in lipid-free areas before and after bilayer indentations to ascertain the formation of a single bilayer. The absence of artifacts or debris on the tip were assessed by the lack of any force-distance step on both trace and retrace curves.

## Supplementary information

Supplementary information.
