# Supplementary material for: C24:0 and C24:1 sphingolipids in cholesterol-containing, five- and six-component lipid membranes
Source: Sci Rep. 2020 Aug 24;10:14085. doi: 10.1038/s41598-020-71008-8 (PMC7445262; doi:10.1038/s41598-020-71008-8)
Supplement: Supplementary file 1 — Supplementary information. [file 41598_2020_71008_MOESM1_ESM.docx]

**SUPPLEMENTARY MATERIAL**

**C24:0 and C24:1 sphingolipids in cholesterol-containing, five- and six-component lipid membranes**

**Emilio J. González-Ramírez^†§*^, Aritz B. García-Arribas^†§*^, Jesús Sot^†§^, Félix M. Goñi^†§^ and Alicia Alonso^†§^.**

^†^ Instituto Biofisika (CSIC, UPV/EHU), 48940, Bilbao, Spain.

^§^ Departamento de Bioquímica, University of the Basque Country (UPV/EHU), 48940, Bilbao, Spain.

**
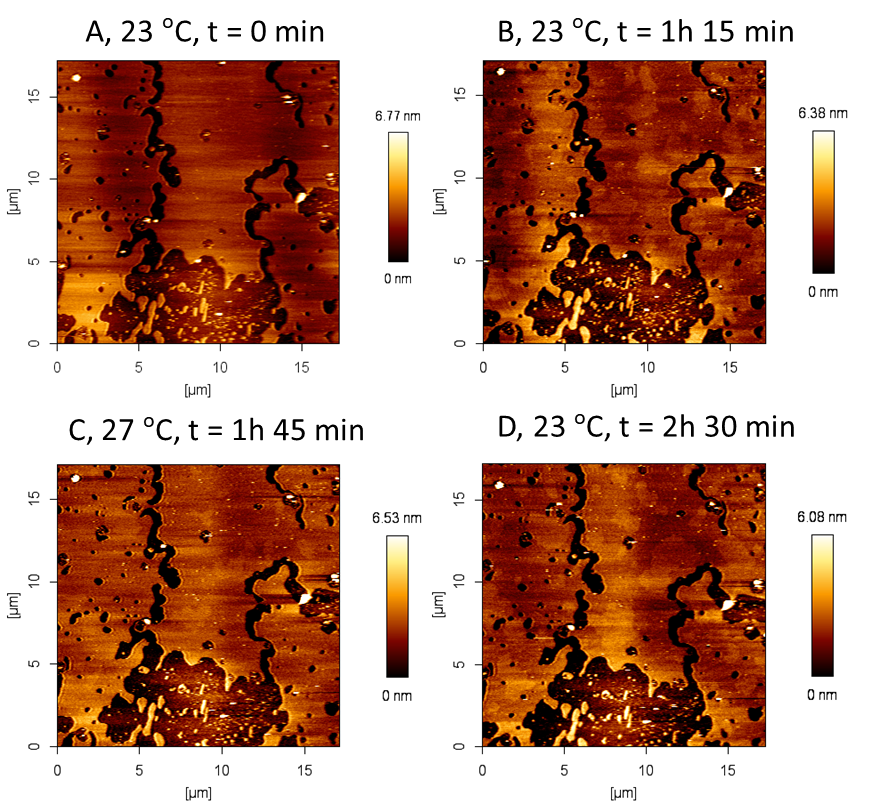
**

**Figure S1. Bilayer dynamism in the absence of Cer: AFM images of SPBs of DOPC:lSM:nSM:Chol (2:0.5:0.5:1).** Domains are not initially seen in our first scan (A) but after 1h they appear spontaneously (B). A small increase in T slightly affects the domains reducing their size but they are still visible (C). When the system returns to room T, domains are visible and their area grows (D). This points to a time-dependent equilibrium in the A-to-B step.


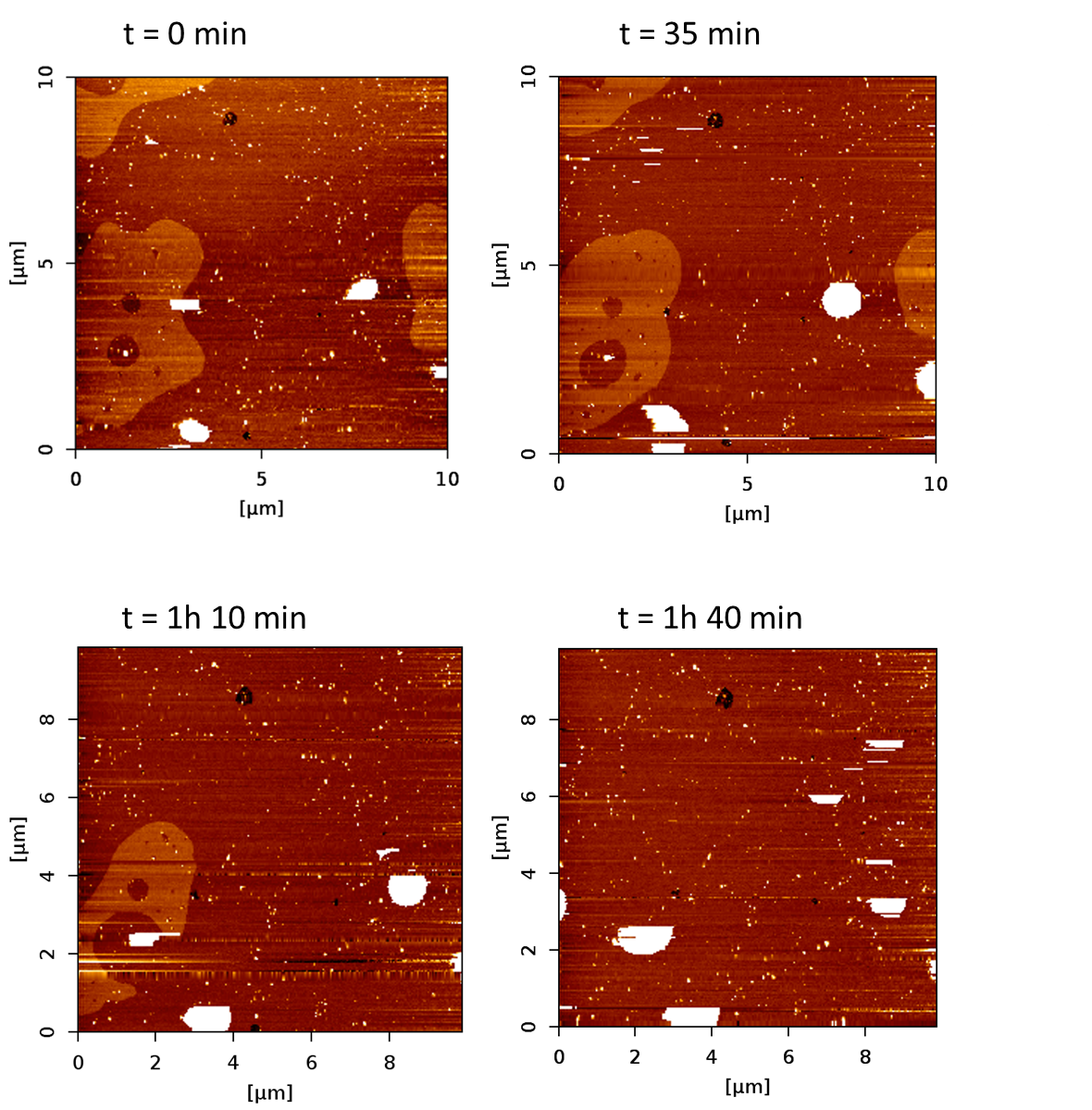


**Figure S2.** **Bilayer dynamism in C24:0 + C24:1 sphingolipid samples containing Cer: another representative example.** AFM images of SPBs of DOPC:nSM:Chol:lCer:nCer (from the same SPB as depicted in Fig. 7C). This example shows that domains completely disappear over time.

**
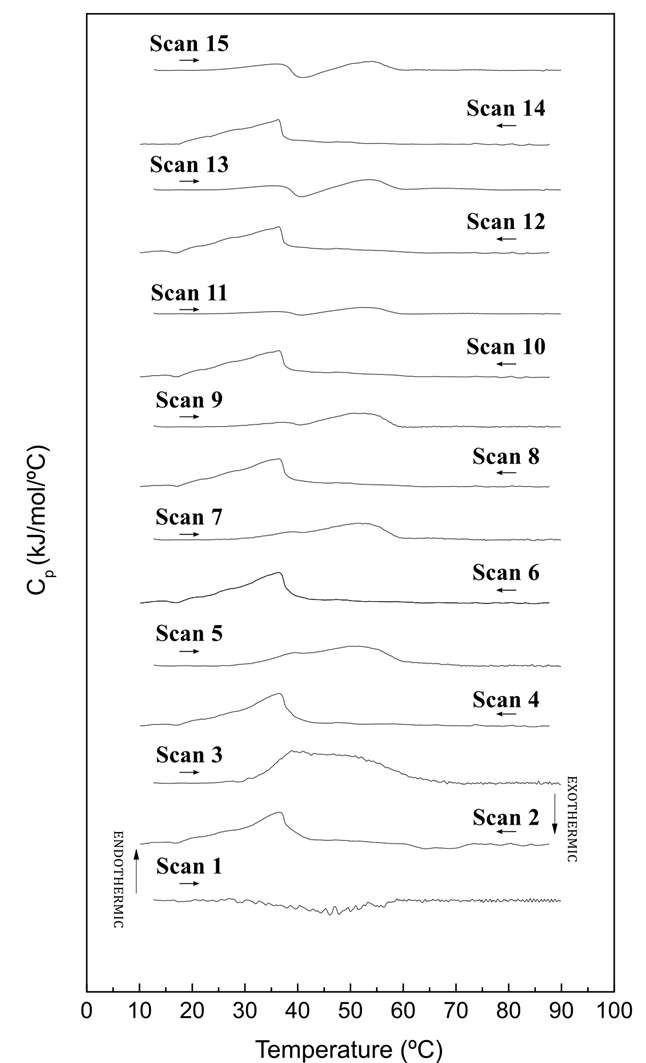
**

**Fig. S3.** Eight successive heating and cooling scans of a sample containing DOPC/24:1SM/ Chol (2:1:1) + 15% 24:0 Cer + 15% 24:1 Cer. Arrows: 4 kJ/^0^C x mol.

**Table S1**. **Height differences between fluid and gel phases in SPBs.** These results have been calculated from cross-section analysis of the AFM images for each sample. Results are shown as mean ± SD of n = 25 - 50 cross-section results.

|  |  | **Fluid/gel phase height difference (nm)** |
| --- | --- | --- |
| DOPC/lSM/Chol | + lCer | 1.82 ± 0.17 |
| DOPC/lSM/Chol + nCer | | 0.97 ± 0.12 |
| DOPC/lSM/Chol + lCer + nCer | | 1.00 ± 0.15 / 1.91 ± 0.19 |
| DOPC/nSM/Chol + lCer | | 1.67 ± 0.24 |
| DOPC/nSM/Chol + nCer | | 1.80 ± 0.24 |
| DOPC/nSM/Chol + lCer + nCer | | 1.01 ± 0.12 |
| DOPC/lSM/nSM/Chol + lCer | | 1.59 ± 0.20 |
| DOPC/lSM/nSM/Chol + nCer | | 0.92 ± 0.15 |
| DOPC/lSM/nSM/Chol + lCer + nCer | | 1.09 ± 0.22 |
